# Co-creation of a school-based sexual health education intervention in Italy

**DOI:** 10.1093/eurpub/ckac129.741

**Published:** 2022-10-25

**Authors:** A Chinelli, M Farinella, L Rancilio, MC Salfa, A Cellini, M Ubbiali, E Torri, A Camposeragna, D Martinelli

**Affiliations:** 1 University of Pisa, Pisa, Italy; 2 Circolo di Cultura Omosessuale Mario Mieli, Rome, Italy; 3 Caritas Ambrosiana, Milan, Italy; 4 National Institute of Health, Rome, Italy; 5 Università La Sapienza, Rome, Italy; 6 Università di Foggia, Foggia, Italy; 7 Università di Verona, Verona, Italy; 8 Coordinamento Nazionale Comunità di Accoglien, Rome, Italy

## Abstract

**Introduction:**

School-based sexuality education (SBSE) is the most effective way to positively impact young people's behaviour and attitude towards sexuality. This study describes the development of a SBSE pilot activity targeting lower secondary schools (LSS) within the context of EduforIST project funded by the Italian Ministry of Health.

**Methods:**

A desk review was carried out to collect information about national policies, international literature and guidelines on SE and STIs prevention. An online survey was developed to collect information on SBSE activities implemented in Italy during 2016-2020. Focus groups among project partners and open consultations with a multisectorial expert advisory board were organised. SBSE pilot activity was developed by an interdisciplinary team of pedagogists, public health and SE experts, educators.

**Results and discussion:**

The SBSE was structured in: a) 5 interactive interventions of 2 hours each with students (4 theoretical and practical modules; 1 final intervention for students-led discussion and evaluation); b) introductory and closing meetings with teachers and with parents. The modules addressed the following dimensions: changes in adolescence; emotions and relationships; sexual identities and diversity; sexual consent, STIs/pregnancy prevention, sexual health services. Additional materials were developed: a. pre/post evaluation tools for educators and students; b. pre-implementation checklist for schools; c. materials for teachers’ and parents’ engagement before/after the SBSE activity.

**Conclusions:**

This pilot activity represented a first step towards the development of a standardised, evidence-based and comprehensive approach to SBSE, for future implementation across the country.

